# Mitochondrial Syndromes Revisited

**DOI:** 10.3390/jcm10061249

**Published:** 2021-03-17

**Authors:** Daniele Orsucci, Elena Caldarazzo Ienco, Andrea Rossi, Gabriele Siciliano, Michelangelo Mancuso

**Affiliations:** 1 Unit of Neurology, San Luca Hospital, 55100 Lucca, Italy; elenacaldarazzoienco@gmail.com; 2 Medical Affairs and Scientific Communications, 1260 Nyon, Switzerland; molezzano.1@libero.it; 3 Department of Clinical and Experimental Medicine, Neurological Clinic, University of Pisa, 56126 Pisa, Italy; g.siciliano@unipi.it (G.S.); m.mancuso@unipi.it (M.M.)

**Keywords:** CPEO, leber, Leigh syndrome, MELAS, MERRF, mitochondrial myopathy, MNGIE, mtDNA, NARP, PEO

## Abstract

In the last ten years, the knowledge of the genetic basis of mitochondrial diseases has significantly advanced. However, the vast phenotypic variability linked to mitochondrial disorders and the peculiar characteristics of their genetics make mitochondrial disorders a complex group of disorders. Although specific genetic alterations have been associated with some syndromic presentations, the genotype–phenotype relationship in mitochondrial disorders is complex (a single mutation can cause several clinical syndromes, while different genetic alterations can cause similar phenotypes). This review will revisit the most common syndromic pictures of mitochondrial disorders, from a clinical rather than a molecular perspective. We believe that the new phenotype definitions implemented by recent large multicenter studies, and revised here, may contribute to a more homogeneous patient categorization, which will be useful in future studies on natural history and clinical trials.

## 1. Background

Mitochondrial diseases are a particularly complex group of disorders caused by impairment of the electron transport chain (or respiratory chain) in the mitochondria. Phenotypes may range from pure myopathy to multi-systemic disorders, having a wide-ranged age at onset, severity, and progression. The genetic defect can be located in the mitochondrial DNA (mtDNA) or the nuclear genome (nDNA). Mitochondrial disorders due to mtDNA mutations are peculiar in human genetics, following different inheritance laws than nuclear genes. MtDNA large-scale single deletions are sporadic and usually not inheritable. MtDNA point mutations are inherited according to the rules of mitochondrial genetics (maternal inheritance, mitotic segregation, heteroplasmy, and threshold effect) [1].

“Polyplasmy” means that each cell contains multiple copies of mtDNA. The term “heteroplasmy” refers to the coexistence of two populations of mtDNA, normal and mutated, while the term homoplasmy describes mtDNA copies identical one to another in healthy individuals. Mutated mtDNA causes dysfunction when present in tissue in a given percentage (“threshold effect”), impairing the oxidative metabolism, varying from cell to cell according to the relative dependence on oxidative metabolism itself. Differences in mutational load, surpassing the pathogenic threshold in some tissues, but not in others, may contribute to the heterogeneity of phenotypes. Because of the mitotic segregation (random share-out of mutated and non-mutated mitochondria between the daughter cells), the mutation load can change between different generations of cells and, with time, it can either surpass or fall below the pathogenic threshold [1].

The expression of mtDNA mutation may also be influenced by other factors, including gender, mtDNA polymorphisms, nuclear genetic background, and additional mtDNA mutations, as well as gene–environment interactions, including smoke and drugs.

The effects of mutations affecting the respiratory chain are frequently multi-systemic, involving either visual and auditory pathways, heart, central nervous system, and/or skeletal muscle. Therefore, some of the “red flags” for considering a mitochondrial disorder include myopathy with exercise intolerance, axonal neuropathy, eyelid ptosis, ophthalmoparesis, pigmentary retinopathy, optic neuropathy, sensorineural hearing loss, diabetes mellitus, hypertrophic cardiomyopathy, migraine, short stature, and cognitive impairment [1].

Adult patients with mitochondrial diseases have an increased risk of mortality that can be independently predicted by the presence of intraventricular cardiac conduction defects, diabetes, and focal brain involvement [2].

As mitochondrial diseases are the most common form of inherited neurometabolic disorders, with pathogenic DNA variants in nuclear or mitochondrial genomes estimated to affect 1 in 4300 individuals [3], these patients can be diagnosed and managed by a wide range of medical specialists. Therefore, an acute “clinical awareness” about this diagnosis is essential to start a correct diagnostic workup. For this reason, even if mitochondrial disorders may represent a complex field, all physicians should be aware of the basic principles of mitochondrial medicine.

This review will describe the most common syndromic pictures of mitochondrial disorders, from both molecular and clinical perspectives. Apart from our previous papers (mostly based on the large database of the Italian Network of Mitochondrial Disorders, supported by Telethon, with 1467 patients included from 2010 to 2016; mean age at onset 23.4 ± 19.5 years, age at last evaluation 39.2 ± 21.7 years, disease duration 15.8 ± 14.2 years; childhood onset (before age 16) 43.8%; males 48% [4]) and their references, we searched PubMed until 9 March 2021 for relevant articles in the last ten years (Table 1). The most typical of these syndromes are summarized in Table 2.

## 2. Clinical Pictures

### 2.1. Isolated Mitochondrial Ataxias

Ataxia is characterized by incoordination or imbalance (e.g., truncal ataxia, gait ataxia) of a limb while executing a task (dysmetria), and is usually caused by dysfunction of the cerebellum or its connections. However, a peripheral sensory involvement may also cause ataxia, and the neurological examination can lead to a correct distinction between the two conditions. Furthermore, cerebellar ataxia is frequently associated with other signs of cerebellar dysfunction, including abnormal eye movements, dysmetria, kinetic tremor, dysarthria, and/or dysphagia.

Ataxia is one of the clinical symptoms of a few well-defined mitochondrial syndromes, including Kearns–Sayre syndrome (KSS); maternally inherited Leigh syndrome (MILS); myoclonic epilepsy (or encephalopathy) with ragged red fibers (MERRF); and neuropathy, ataxia, and pigmentary retinopathy (NARP), and may be present in undefined mitochondrial encephalopathies [5].

Even if cerebellar ataxia can be the onset symptom of one of these mtDNA-associated disorders, discussed in-depth in the following paragraphs, isolated ataxia may also be due to autosomal recessive defects in intergenomic signaling. These include *POLG*-related diseases (i.e., ataxia-myopathy syndrome [6]; “mitochondrial recessive ataxia syndrome”, or MIRAS [7]; ataxia with ocular myopathy and, more rarely, with psychiatric comorbidities or epilepsy [8]) and some *C10orf2* (Twinkle) mutations, which can cause infantile-onset spinocerebellar ataxia (IOSCA), a severe neurodegenerative disorder characterized by progressive atrophy of the brainstem, cerebellum, and spinal cord and sensory axonal neuropathy [9]. Ataxia can be the first symptom in rare patients with autosomal dominant optic atrophy (ADOA) due to *OPA1* gene mutations [10].

Among the above described forms, *POLG* mutations are likely the most frequent. A recent multinational, retrospective study on 155 patients with *POLG* variants showed that the clinical features form a continuum. Interestingly, ataxia was the major feature (90%) for *POLG* patients with onset between 12 and 40 years, and the second feature (58%) after ocular myopathy in older subjects [11].

Furthermore, isolated cerebellar ataxia is one of the typical features of autosomal recessive coenzyme Q10 deficiencies, and coenzyme Q10 biosynthetic genes (e.g., *CABC1*) are the primary candidate for molecular analysis in patients with progressive cerebellar ataxia and atrophy on imaging studies [12]. Coenzyme Q10 deficiency is a treatable condition; therefore, its diagnosis is essential.

### 2.2. Isolated Mitochondrial Myopathies

Muscle symptoms, including proximal limb weakness, muscle fatigue, exercise intolerance, and pain, are common in mitochondrial disorders. Many patients with mitochondrial disorders may present with an isolated myopathy. “Primary mitochondrial myopathies” have been defined as disorders leading to defects of oxidative phosphorylation predominantly affecting skeletal muscle [13] (and not fulfilling the criteria for other, more complex clinical syndromes).

**Figure 1:**
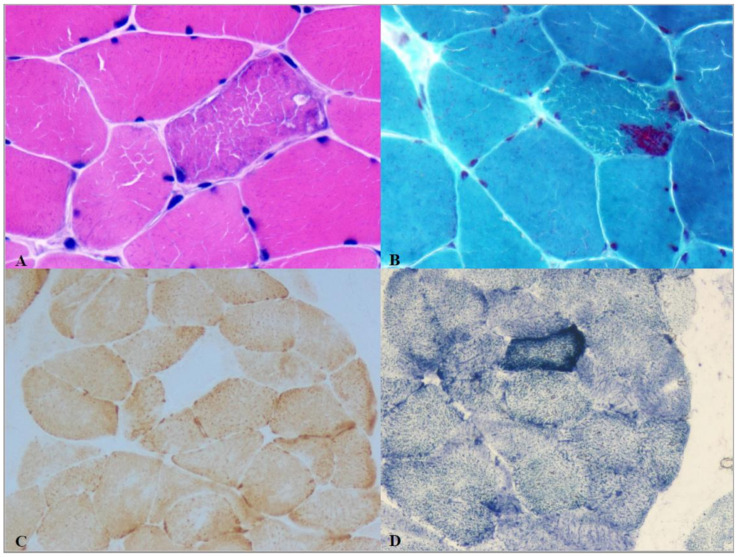
Histological findings in mitochondrial myopathy. (**A**,**B**) Ragged red fiber (Hematoxylin–Eosin and Gomori’s staining, respectively, at the center of each panel), (**C**) cytochrome c oxidase (COX)-negative fibers (“white” fibers, COX staining), and (**D**) “Ragged blue” fibers (SDH (succinate dehydrogenase) staining).

Fatigue and exercise intolerance are elusive symptoms. In the database of the Italian Network of Mitochondrial Disorders, more than 20% of mitochondrial patients reported exercise intolerance. Creatine kinase (CK) levels were increased in about one-third of the patients with this symptom. Moreover, all the other “myopathic” signs (muscle pain, muscle wasting, cardiomyopathy, and eyelid ptosis/ophthalmoparesis) were associated with exercise intolerance. Ragged red fibers and, most importantly, cytochrome c oxidase (COX)-negative fibers (Figure 1) were more frequently present in exercise intolerants [14]. Other specifically designed studies confirmed that fatigue was common among patients with mitochondrial disease and that the severity of fatigue could correlate with the severity of disease [15].

A recent cohort study described the baseline features of 118 adult patients with primary mitochondrial myopathy. The 6-minute walk test (6MWT), timed water swallow test (TWST), timed up-and-go test (x3) (3TUG), five-times sit-to-stand test (5XSST), and test of masticating and swallowing solids (TOMASS) were used as functional outcome measures. The West Haven-Yale Multidimensional Pain Inventory and Fatigue Severity Scale were assessed as patient-reported outcome measures. Some biomarkers (FGF21, lactate, GDF15, and CK) were also measured. Interestingly, all the functional outcome measures were significantly out of normal ranges and correlated with perceived fatigue and pain severity. FGF21 and GDF15 were higher in the patients compared with controls, but were not predictors of disease severity [16].

The mosaic pattern of COX-negative fibers and ragged-red fibers interspersed among normal-appearing fibers is the histologic hallmark of mitochondrial myopathy (even if it must be noted that COX-negative fibers may be lacking, e.g., in some cases of mitochondrial cytochrome B mutations). The biopsied muscle is the optimal tissue for molecular genetic studies of mtDNA to be used to test the underlying mtDNA mutations, as well as in samples lacking the classic histologic findings. Enzyme histochemistry and functional assays of mitochondrial enzyme activity can be performed using both fresh or frozen tissue muscle [17]. Interestingly, the presence of these histopathological features was associated with ophthalmoplegia, weakness, myopathic facies, and exercise intolerance in children with a mitochondrial disorder [18].

As well as for cerebellar ataxia, one must consider the possibility of a treatable coenzyme Q10 deficiency in every patient with isolated mitochondrial myopathy. Primary coenzyme Q10 deficiencies must be treated with high-dose coenzyme Q10, whereas rare secondary deficiencies due to *ETFDH* mutations may benefit from riboflavin treatment.

### 2.3. Leigh Syndrome and NARP

The most common pediatric clinical presentation of mitochondrial disease is Leigh syndrome.

Leigh syndrome has a wide biochemical and molecular profile. Numerous mutations in about 100 genes across both nuclear and mitochondrial genomes are known to cause this disorder. Even if the diagnostic criteria have not yet been redefined following the expanding knowledge of molecular aetiologies [19], Leigh syndrome is thought to affect about 1 in 40,000 births. Although this disorder is characterized by marked clinical and genetic heterogeneity, the typical neuropathological features are consistent and include focal, necrotizing lesions of the basal ganglia, cerebellum, diencephalon, and/or brainstem, leading to a progressive decline of neurological function. The clinical hallmarks include psychomotor delay or regression, hypotonia, tremor, weakness, truncal ataxia, and lactic acidosis [20]. Dystonia is often associated with Leigh syndrome too [21].

The typical clinical onset occurs in the first 2 years and patients die at about 3 years of age. Delayed development is the first manifestation in most patients. Motor weakness and ataxia may be more common in children with onset after 2 years, with a less severe clinical course [20].

Recently, a systematic review reported that the most common features of Leigh syndrome were elevated blood and/or cerebrospinal fluid (CSF) lactate (72%), developmental retardation (57%), hypotonia (42%), respiratory dysfunction (34%), epileptic seizures (33%), poor feeding (29%), and weakness (27%). About 80% of the patients had deficiencies of the respiratory chain enzyme complexes (isolated complex I deficiency in 35% of cases), whereas 38% had nDNA mutations and 32% had mtDNA mutations [22].

MILS is the mtDNA-associated “maternally inherited Leigh syndrome”, presenting generally during the first year of life with ataxia, motor retardation, neuropathy, hypotonia, epileptic seizures, myoclonus, pigmentary retinopathy, optic atrophy, lactic acidosis, and psychomotor regression. It is usually associated with point mutations in the gene encoding APTase6 (a subunit of the complex V of the respiratory chain, or ATP synthase), especially the m.8993T>G. If the percentage of mutant mtDNA is between 70% and 90%, the clinical presentation is the NARP syndrome, a more benign late-onset syndrome characterized by ataxia, neuropathy, and pigmentary retinopathy [5]. Even if the mtDNA background seems to play an important role in modulating the biochemical defects and clinical outcome in NARP/MILS, relevant phenotypic differences have been reported between family members too [23]. Interestingly, a few MILS patients may have an unexpected recovery of the disease, turning to a more benign NARP phenotype in adult life [24].

### 2.4. MELAS: Mitochondrial Encephalopathy with Lactic Acidosis and Stroke-Like Episodes

“Mitochondrial encephalopathy with lactic acidosis and stroke-like episodes” (MELAS) is a mitochondrial syndrome having a strong, not exclusive, association with the m.3243A>G mutation of the mtDNA. MELAS is characterized by subacute stroke-like episodes linked to seizure activity [25] and is frequently associated with diabetes and lactic acidosis.

**Figure 2:**
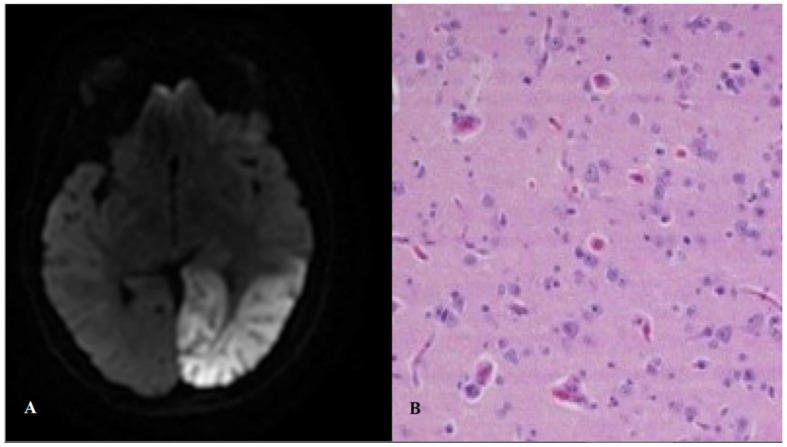
Stroke-like lesion. Magnetic resonance (**A**) and histopathological (**B**) findings. Panel B shows a brain biopsy from a MELAS (mitochondrial encephalopathy with lactic acidosis and stroke-like episodes) patient, with “red neurons” and spongiosis with reactive astrocytosis in the cortex and the white matter (Hematoxylin–Eosin).

A stroke-like episode has an acute or subacute presentation, and is caused by focal brain dysfunction, typically presenting before the fourth decade of life. The severity of the neurological disability (e.g., aphasia, apraxia, hemianopia, cognitive impairment, cortical blindness, psychosis, and weakness) depends on the extent of temporal, parietal, and/or occipital lobe involvement. Neuroimaging permits the discrimination between a stroke-like episode and an ischaemic infarct by evidencing the extension of the lesion(s) beyond a well-defined vascular territory. These lesions affect especially the temporal and occipital lobes and can be bilateral (Figure 2). Seizure activity is frequently detected by electroencephalography [26].

Progressive accumulation of neurological deficits is the result of recurrent attacks of stroke-like episodes followed by symptomatic remission. Increased cerebral blood flow was reported in stroke-like episodes at the attack stage, while decreased blood flow may be typical of the recovery stage. Furthermore, the reduction of spontaneous brain activity beyond the areas of stroke-like lesions has been identified in a recent functional magnetic resonance imaging study; this phenomenon downwards from the attack stage to remission in MELAS patients [27].

A recent systematic review showed that patients with MELAS had significantly more electrocardiography and echocardiography (especially left ventricular hypertrophy) abnormalities than patients with other mitochondrial phenotypes. A common and well-described cardiac complication overrepresented in MELAS was Wolff–Parkinson–White syndrome (WPW) [28]. The worst prognosis can be independently predicted by a personal history of seizures and left ventricular hypertrophy in these patients [29].

The Italian Network studied the phenotypes associated with the m.3243A>G “MELAS” mutation in 126 patients, confirming the high clinical heterogeneity of this mutation. Hearing loss and diabetes were the most frequent clinical features, followed by stroke-like episodes. “MIDD” (maternally-inherited diabetes and deafness) and “PEO” (progressive external ophthalmoplegia) were nosographic terms not relevant for m.3243A>G carriers; these patients, supposed to have a less severe disease, were susceptible to multisystem complications (e.g., heart involvement and stroke-like episodes). The “MELAS” acronym defines patients with stroke-like episodes and histological, molecular, and/or biochemical evidence of mitochondrial disease. Moreover, the male gender was identified as a possible predictor for stroke-like episodes in Italian m.3243A>G carriers [30]. However, this observation has not yet been confirmed in other patients’ cohorts, and longitudinal studies are still needed.

### 2.5. MERRF: Mitochondrial Encephalopathy with Ragged Red Fibers

Myoclonus is a succession of repeated brief shock-like jerks, often non-rhythmic, due to unexpected, rapid, involuntary relaxation or contraction of one or more muscles. It can be classified as focal, multifocal, or generalized according to its distribution and, by provoking factors, as spontaneous or reflex [31]. Rarely, myoclonus is part of a group of symptomatic generalized epilepsies, defined as progressive myoclonus epilepsies, with a debilitating course and poor outcome [32]. The most common progressive myoclonus epilepsy is MERRF (“myoclonic epilepsy with ragged red fibers”).

MERRF is a mitochondrial syndrome characterized by seizures, myoclonus, and ataxia. MERRF patients can have different clinical onset and they are carriers of various mtDNA point mutations, the most frequent of which is the m.8344A>G change in the tRNA lysine. The Italian Network of Mitochondrial Disorders observed that the majority of 42 patients carrying this mutation did not present the MERRF typical clinical picture. Myoclonus was present in 20% of the patients, whereas myopathic signs and symptoms, hearing loss, generalized seizures, eyelid ptosis, and multiple lipomatosis were the most common features. Some m.8344A>G mutation carriers did not show any symptoms. Myoclonus was more strictly associated with ataxia than with generalized seizures, suggesting that MERRF could be better defined as myoclonic ataxia rather than myoclonic epilepsy [33].

Myoclonus is an uncommon feature in mitochondrial diseases (for instance, it was present in less than 5% of the subjects included in the database of the Italian Network) not strictly linked to MERRF syndrome, being detected in other typical mitochondrial encephalopathies like MELAS and Alpers syndromes. While most of the patients with other well-defined mitochondrial encephalopathies had generalized epileptic seizures and a small percentage had ataxia, these percentages are reversed in MERRF subjects [34]. Therefore, the term “myoclonic epilepsy” seems inadequate and potentially misleading. Considering that, in many previous articles, the meaning of “MERRF” is unclear (i.e., in many instances, it is not specified if it has been used to denote mitochondrial patients with this phenotype or subjects with the m.8344A>G mutation independently from the clinical picture), the practical definition of “MERRF” as, the “mitochondrial syndrome where myoclonus is the prominent clinical feature, and which does not meet the criteria of other well-defined mitochondrial encephalopathic syndromes (e.g., MELAS, Leigh or Alpers syndromes)”, could be more appropriate. The acronym “MERRF” is probably too entrenched to be eliminated; therefore, our group proposed its definition as “myoclonic encephalopathy with ragged red fibers”. Prospective, well-designed studies are needed to have a complete picture of the natural history of this disease [34].

### 2.6. Mitochondrial Cardiomyopathies

About 30% of adults with any mitochondrial disease have electrocardiographic and/or echocardiographic abnormality. More than one-third of these patients reveal late gadolinium enhancement, associated with myocardial fibrosis, at cardiac magnetic resonance. Cardiac involvement has been associated with worst prognosis, highlighting the need for effective therapies able to protect against cardiac damage [35]. For instance, a retrospective study reported that intraventricular conduction block, left ventricular hypertrophy, premature ventricular complexes, and diabetes were independent predictors of major adverse cardiac events in adults with mitochondrial diseases [36].

A study on adults with limited forms of mitochondrial myopathy revealed a myocardial wall remodeling characterized by early left ventricular systo-diastolic function abnormalities and increased fibrosis. Left ventricular dysfunction needs to be properly managed, apart from cardiac conduction defects such as bundle branch blocks, prolonged intraventricular conduction time, and complete atrioventricular block [37]. Furthermore, cardiac muscle involvement, often present as hypertrophic cardiomyopathy, may occur in 20% to 40% of patients with the m.3243A>G mutation [26] and may be associated with other mutations as well. Recently, pathogenic variants in glutamyl-tRNA Gln amidotransferase subunits, revealing cardiomyocytes with massive mitochondrial proliferation in post-mortem examinations, have been specifically linked to a lethal mitochondrial cardiomyopathy disorder [38].

Cardiomyopathy occurs in approximately one-third of children with mitochondrial diseases, having from asymptomatic to severe (with arrhythmias, heart failure, and sudden cardiac death) clinical presentations. More than 50% of cases have hypertrophic cardiomyopathy; however, mitochondrial cardiomyopathies might also present in the restrictive, dilating, left ventricular non-compaction and/or histiocytoid forms [39]. Heart disease was observed to be more common in male children with specific mitochondrial conditions (Barth syndrome, *TMEM70* mutations, and MELAS). Non-compaction, hypertrophic, and dilated cardiomyopathies were the prevalent conditions, while pulmonary arterial hypertension was less frequent [40].

Therefore, complete cardiologic evaluation must represent a standard assessment in the management of children and adults when a mitochondrial disease is known or suspected. Furthermore, the diagnosis of mitochondrial cardiomyopathy should be considered when evaluating patients with any cardiomyopathy of unknown origin.

### 2.7. Mitochondrial Neuropathies

Mitochondrial disorders involving the peripheral nervous system are well known. However, the exact prevalence of peripheral neuropathy in mitochondrial disorders is still unclear. In the database of the Italian Network of Mitochondrial Disorders, the prevalence of clinically apparent peripheral neuropathy was 12.4%. *POLG* (nuclear gene) mutations could cause a potentially axonal/mixed, painful, mainly sensory polyneuropathy; *TYMP* (nuclear gene) mutations could lead to a sensory-motor demyelinating polyneuropathy; *SURF1* (nuclear gene) mutations were associated with a demyelinating/mixed sensory-motor polyneuropathy. The only mtDNA mutation potentially associated with neuropathy was the m.8993T>G (or T>C) change (“NARP” mutation), underlying to an axonal, mainly-sensory polyneuropathy [41].

Even if neuropathy is not the most common feature of mitochondrial diseases, it can have a strong impact on the quality of life of the affected patients. Furthermore, diagnosing peripheral neuropathy may support and address the molecular analysis.

Mitofusin-2 (*MFN2*) neuropathy must be cited in addition to these “conventional” mitochondrial disorders. The causal relationship between the hereditary axonal neuropathy Charcot–Marie–Tooth disease type 2A (CMT) and *MFN2* gene mutations were described at the beginning of the XXI century [42]. MFN2 dysfunction preferentially impacts peripheral nerves by impairing mitochondrial fusion, transport, and survival. *MFN2* mutations comprise more than 5% of all CMT cases. These patients have been exhibiting, frequently from childhood, loss of sensory and motor nerve function in the distal limbs, but some patients show proximal limb weakness due to myopathic involvement. Interestingly, while CMT2A is almost always heterozygous, with autosomal dominant inheritance, bi-allelic *MFN2* mutations may lead to multiple lipomatosis [42], which is another rare, but well-known feature of mitochondrial dysfunction (more commonly associated with the m.8344A>G “MERRF” mutation).

### 2.8. Mitochondrial Ocular Myopathies (I): PEO and PEO Plus

Ocular myopathy, also defined as progressive external ophthalmoplegia (PEO) when this is the main clinical trait, is the most frequent mitochondrial phenotype. PEO can start with progressive bilateral eyelid ptosis and ophthalmoparesis at different ages. Ptosis is typically the earliest and most significant facet, often leading to the gradual acquisition of a chin-up compensatory head position [43]. Intermittent, mainly horizontal, diplopia can be present in about one-third of the patients. CK levels can range from normal to mildly or moderately increased [44].

Even if there is still a strong need for a more homogeneous patient categorization, the term “pure PEO” has been suggested to identify the patients with isolated ocular myopathy, whereas “PEO plus” identifies those with ocular myopathy and other features of neuromuscular involvement. The patients with ocular myopathy and evidence of structural central neurological dysfunction should not be defined as having “PEO”, but they are affected with a more complex mitochondrial encephalomyopathy [45].

Ocular myopathy, with eyelid ptosis and/or ophthalmoparesis, is the most common trait of mitochondrial disorders, being present in half of the patients with a confirmed mitochondrial disorder. In the large database of the Italian Network, ocular myopathy was more strictly associated with the molecular finding of an mtDNA single deletion or a *POLG* mutation. Patients with *ANT1*, *Twinkle*, and *TYMP* mutations show an increased frequency of ocular myopathy too, while carriers of *OPA1* mutations and all the mtDNA point mutations showed a lower prevalence. Muscle weakness (43%), exercise intolerance (23%), muscle wasting (18%), hearing loss (15%), and swallowing impairment (15%) were the symptoms more frequently associated with PEO plus [45].

PEO patients also complain of severe fatigue, pain, and depression contributing to disease impact, causing a worsened quality of life [46]. Swallowing impairment and decreased respiratory muscle strength can be present and should be actively detected. Obstructive apneas may also be present. Furthermore, a study performed on 23 PEO patients reported decreased respiratory muscle strength, both inspiratory and expiratory. Therefore, periodic assessments of respiratory functions in PEO patients are needed [47].

### 2.9. Mitochondrial Ocular Myopathies (II): Pearson Syndrome and “Kearns–Sayre Spectrum”

Excluding PEO, single deletions of mtDNA have been associated with two complex phenotypes: Pearson syndrome (a super rare disease characterized by pancreatic insufficiency and refractory sideroblastic anemia) and Kearns–Sayre syndrome (KSS, ptosis/ophthalmoparesis associated with pigmentary retinopathy and other specific diagnostic criteria), in a continuum of clinical phenotypes with a large number of variants [48].

Pearson syndrome is a multisystem disorder due to the mitochondrial respiratory chain deficit characterized by exocrine pancreatic insufficiency and sideroblastic anemia from the first year of life, with a poor prognosis. The rare children who survive may progress to KSS later in life [49].

Pearson syndrome represents a dramatic, but numerically limited subgroup of mitochondrial patients (<3%). Exocrine pancreatic insufficiency was uncommon in the cohort of the Italian Network; in fact, it was less frequent than increased liver enzymes, lactic acidosis, pancytopenia, failure to thrive/short stature, hypotonia, ophthalmoparesis, ptosis, diabetes mellitus, and kidney involvement [48]. These findings are consistent with the results of a study on 34 pediatric single-deletion patients, where renal impairment, anemia, and endocrine disturbance were the most frequent extra-neurological traits. Survival analysis revealed significantly higher mortality in patients with hematological features (Pearson syndrome) than in others [50].

KSS is traditionally defined by the presence of PEO and pigmentary retinopathy. Furthermore, the onset occurs before 20 and at least one characterizing symptom (cardiac conduction block, cerebellar ataxia, CSF protein levels > 0.1 g/L) must be present [51]. However, these criteria present some limitations: (I) the age limit of 20 years is arbitrary; (II) CSF examination has very limited use in the clinical practice; and (III) many patients with ptosis and/or ophthalmoparesis due to an mtDNA single deletion do not fulfill the criteria for KSS nor “pure” PEO.

Based on the data from the Italian Network, our group has proposed new criteria for the “KSS spectrum”, where classic KSS represents the most severe extreme of this category. The “KSS spectrum” criteria represent an extension of the old KSS criteria, including the multisystem clinical features that were strongly associated with traditional KSS features, thus being able to predict their presence or later development. These features associated with the signs and symptoms defining KSS (which may, therefore, become predictive) include tremor, hearing loss, cognitive involvement, failure to thrive/short stature, and cardiomyopathy [48].

While, when using the “classic” criteria, it was possible to classify only 66.2% of the patients, the Italian Network classified nearly all patients (225/228, 98.7%) with the “new” ones: 65% PEO (147/228), 32% “KSS spectrum” (72/228), and 3% Pearson syndrome (6/228). These new criteria distinguished between two “natural” groups of patients (the arbitrary parameter of onset before age 20 was excluded). “KSS spectrum” define a multisystem disease with a mean age at onset of 19 years, a more severe muscular impairment (with muscle wasting and weakness), frequently having abnormal brain magnetic resonance imaging (MRI), and worst prognosis. PEO is a prominently myopathic disease with a mean age at onset of 26 years, with cerebral MRI being frequently normal and better prognosis [48].

### 2.10. MNGIE: Mitochondrial Neurogastrointestinal Encephalomyopathy

Mitochondrial neurogastrointestinal encephalomyopathy (MNGIE) is a very rare autosomal recessive disease, caused by thymidine phosphorylase (TP) deficiency and *TYMP* mutations, which usually leads to death in early adulthood. Symptoms include eyelid ptosis and ophthalmoparesis, progressive gastrointestinal dysmotility, cachexia, peripheral neuropathy, and leukoencephalopathy [52]. Mitochondrial dysfunction is associated with thymidine accumulation in plasma and tissues. Therapeutic options aimed at counterbalance the nucleoside accumulation are available, and TYMP-associated MNGIE is one of the few mitochondrial diseases potentially susceptible to treatments, discussed elsewhere [53]. The available options include liver transplantation, allogeneic hematopoietic stem cell transplantation, and carrier erythrocyte entrapped thymidine phosphorylase therapy. Further promising therapies are expected in the near future [52].

The mean age at onset is 18 years. Gastrointestinal symptoms include diarrhea, abdominal pain, vomiting, pseudo-obstruction, weight loss, and cachexia. The typical neurological features are eyelid ptosis and ophthalmoparesis, polyneuropathy, hearing loss, and leukoencephalopathy. Further evidence-based information on MNGIE diagnosis, prognosis, and treatment is available in a very recent position paper [53].

### 2.11. Non-Syndromic Hearing Loss (NSHL)

Hearing impairment is a common clinical feature, either as an isolated manifestation (i.e., NSHL) or as part of a disease spectrum (e.g., MELAS).

Mitochondrial NSHL is a good example of how mitochondrial environmental (toxic) and genetic factors interact to determine the phenotype of each patient. Because of their peculiar prokaryotic origins, mitochondria are possibly vulnerable to several antibiotics that target the bacterial ribosome, especially when certain mutations of the mitochondrial genome are present. The mitochondrial mutations m.1555A>G and m.1494C>T can increase the aminoglycoside drug-binding, making the ribosomal decoding site hyper susceptible to aminoglycoside-induced mistranslation and protein synthesis inhibition [54]. Aminoglycoside may lead to deafness if administered to patients with these mtDNA mutations; the screening test for mtDNA mutations is, thus, recommended before starting the treatment.

### 2.12. Optic Neuropathies (I): LHON

Leber hereditary optic neuropathy (LHON) is a neurodegenerative disorder characterized by subacute, painless, bilateral, and central vision loss that mainly affects healthy young males [55]. The visual prognosis is generally poor, with most patients worsening to 20/200 visual acuity or less. LHON is caused by pathogenic mutations in mtDNA; the m.11778G>A (*MTND4*) is the most common and is associated with poorer outcomes [56]. Even if it is not yet known why LHON mutations clinically affect only some carriers, foveal morphological changes are also present in asymptomatic carriers of the m.11778G>A mutation [57]. A recent meta-analysis of 15 studies analysing the visual function on 695 LHON patients with the m.11778G>A mutation showed that 14.4% of them “recovered” some vision. A younger age at onset (especially <12 years) may lead to a better visual prognosis. Meaningful vision recovery is observed in less than 20% of patients aged ≥15 years [56].

Gender has also been proposed as a risk factor for optic neuropathy in these patients; LHON is a disorder where only 50% of males and 10% of females who carry a pathogenic mutation develop the disease. The protective effect of estrogens has been proposed to in part explain this gender effect [26].

Gene–environment interactions may also have a role. A study identified visual loss in 93% of men who smoked. There was a trend towards increased visual failure with heavy intake of alcohol. Therefore, carriers of an “LHON” mtDNA mutation should be strongly advised not to smoke and to moderate their alcohol intake [58].

### 2.13. Optic Neuropathies (II): Autosomal Dominant Optic Atrophy (ADOA)

Mutated *OPA1* is the most common cause of ADOA. OPA1 protein is involved in several important cellular processes including the stability of the mitochondrial network via its pro-fusion properties. Different from LHON, visual failure in ADOA is frequent (>85%), with a mean age at onset of 8 years. Besides, extra-ocular neurological complications were identified in about 20% of patients (“ADOA plus”) in a large observational study. Bilateral sensorineural deafness, with onset in late childhood and early adulthood, was described as the prominent clinical attribute. In the third decade of life, a combination of ataxia, peripheral neuropathy, myopathy, and ocular myopathy may appear. Spastic paraparesis and multiple sclerosis-like leukoencephalopathies are rarely present. Histochemical and molecular characterization of skeletal muscle biopsies revealed the presence of multiple mtDNA deletions and COX-deficient fibers [10].

### 2.14. Parkinsonisms

Secondary mtDNA rearrangements due to nuclear gene (mostly *POLG)* mutations and mtDNA genetic abnormalities can, sometime, cause parkinsonism. Mitochondrial parkinsonisms do not differ from typical Parkinson’s disease, not allowing an immediate differential diagnosis. Usually, the age at onset is about fifty. A good response to levodopa or dopamine agonists and reduced dopamine uptake in the corpus striatum are well-known attributes. Levodopa-induced motor fluctuations and dyskinesias may also occur. *POLG* gene should be considered in the differential diagnosis of parkinsonisms, and *POLG* mutations should be tested especially in families with autosomal dominant transmission. However, a negative family history does not exclude a possible diagnosis of mitochondrial disease. The diagnosis of mitochondrial parkinsonism should have to be considered when there are other features of mitochondrial dysfunction, such as PEO, neuropathy, myopathy, ataxia, isolated muscle pain, psychiatric disorders, epilepsy, and/or others [59]. *Twinkle* mutations may also cause familial parkinsonism [60].

### 2.15. Pediatric Myocerebrohepatopathies, Including Alpers Syndrome

Alpers syndrome is a severe pediatric encephalopathy associated with liver failure, due to recessive *POLG* mutations [61]. It is typically defined by the classic clinical triad of liver degeneration, seizures, and progressive developmental regression; however, a wide range of clinical expression may occur. Liver involvement may precede or occur after seizure onset; terminal liver failure is common. Seizures can be intractable, with frequent episodes of epilepsia partialis continua or status epilepticus [62].

As children with Alpers syndrome are at high risk of death from status epilepticus or liver failure if exposed to valproate, its use must be avoided in these young patients [54].

Other disorders of mitochondrial maintenance (mtDNA depletion disorders) may manifest with similar phenotypes; i.e., recessive mutations of the Twinkle helicase can also manifest as early encephalopathy with liver involvement, a phenotype reminiscent of Alpers syndrome (which is more commonly linked to *POLG*) [63]. *DGUOK*, *SUCLG1*, and *MPV17* mutations may also present with similar phenotypes, whereas *RRM2B*, *SUCLA2*, and *TK2* are linked to pediatric mtDNA depletion encephalomyopathies without any liver disease [62].

### 2.16. Tumors

Rarely, mitochondrial disorders may present with multiple lipomatosis, a rare disorder characterized by the development of non-encapsulated lipomas (especially in patients with mtDNA mutation in the tRNA-lysine gene) [64], or with tumors of adrenals or sympathetic and parasympathetic paraganglia (i.e., paragangliomas and pheochromocytomas, related to nuclear mutations in the subunits of succinate dehydrogenase gene: *SDHB*, *SDHC*, *SDHD*) [65]. However, most mitochondrial patients are not at increased risk of benign or malignant tumors.

### 2.17. Unspecified Mitochondrial Encephalomyopathies

The clinical pictures described above are the exception in mitochondrial medicine. More complex, unspecified, incomplete, and/or overlap (e.g., MELAS/Leigh) [19] phenotypes may be more frequent.

A very recent registry-based study from North America confirmed that the most common clinical diagnosis was “multi-systemic disorder”, whereas only a minority of mitochondrial patients were diagnosed with a “classical” mitochondrial syndrome such as MELAS or Leigh syndrome, which were the most common (<10% each). These data demonstrate the high variability of genetic, biochemical, and clinical features of mitochondrial patients [66].

Some subjects with a gene mutation that have been historically linked to a typical clinical picture frequently present non-specific phenotypes. For instance, a recent study revealed that more than 50% of patients with *MT-ATP6* mutations do not show a typical MILS/NARP phenotype, but variable nonsyndromic features including neuropathy, ataxia, and learning disability [67].

A rare autosomal recessive condition that may be associated with different encephalomyopathic phenotypes is coenzyme Q10 deficiency. Coenzyme Q10 deficiency has been linked to five major syndromes: (i) encephalomyopathy (associated with recurrent myoglobinuria, muscle ragged red fibers, and brain involvement); (ii) severe infantile multi-systemic disease; (iii) cerebellar ataxia; (iv) Leigh syndrome; and (v) isolated myopathy. Primary coenzyme Q10 deficiency due to mutations in ubiquinone biosynthetic genes (i.e., *CABC1*, *COQ2*, *PDSS1*, *PDSS2*) has been identified in patients with cerebellar and infantile multi-systemic phenotypes. In contrast, secondary coenzyme Q10 deficiency, due to mutations in genes not directly related to ubiquinone biosynthesis (i.e., *APTX*, *BRAF, ETFDH*), has been identified in patients with pure myopathy and cerebellar ataxia [68]. Coenzyme Q10 deficiency is a treatable condition; therefore, it is very important to consider this possible diagnosis. Early treatment with high-dose coenzyme Q10 may dramatically change the prognosis of this disease.

## 3. Conclusions

The development of next generation sequencing has led to a significant improvement in the genetic diagnostic capacity in the last ten years, dramatically expanding the scenario of the genetic basis of mitochondrial diseases [69]. The diagnostic power may improve with “multi-omics” integrative approaches too [70].

A non-invasive, bigenomic (nDNA and mtDNA) sequencing approach (using both whole-exome sequencing and optimized mtDNA analysis to include large deletions) could be the first step in investigating mitochondrial disorders. Muscular biopsy will be limited to unsolved or unclear cases after genetic studies [71]. Single-fiber genetic studies may also be useful in the diagnosis of patients harboring heteroplasmic mtDNA variants of unknown significance [72]. The mitochondrial genome should be sequenced, from muscle DNA, in all unusual neurological syndromes, even without a clear maternal inheritance.

The unique maternal inheritance and multi-copy presence of the mitochondrial genome and the clinical heterogeneity associated with mitochondrial disorders make mitochondrial disorders a remarkably complex group of diseases. Although some syndromic presentations have been associated with specific genetic alterations, the genotype–phenotype relationship in mitochondrial disorders is particularly complex because a single mutation can cause several different clinical syndromes, while each syndrome can be caused by different genetic alterations.

The most typical mitochondrial syndromes are summarized in Table 3. We believe that the new phenotype definitions implemented by recent large multicenter studies may contribute to a more homogeneous patient categorization, to be used in future studies on the natural history of mitochondrial disorders or in clinical trials.

## Figures and Tables

**Table 1:** Pubmed search. The parameters of our search on Pubmed are reported here. Note that the symbol * is the Pubmed wildcard for every possible word beginning or ending.

merrf[Title] OR melas[Title] OR narp[Title] OR leigh[Title] OR leber[Title] OR adoa[Title] OR mitochondrial disease * [Title] OR mitochondrial disorder * [Title] OR mitochondrial myopath * [Title] OR mitochondrial encephalomyopath * [Title] OR kearns-sayre[Title] OR progressive external ophthalmoplegia[Title] OR iosca[Title] OR miras[Title] OR alpers[Title] OR coenzyme Q10 deficienc * [Title] OR pearson[Title] OR mngie[Title] OR nshl[Title] OR mitochondrial encephalopath * [Title] OR mitochondrial cardiomyopath * [Title]
**Filters applied: in the last 10 years, English**

**Table 2:** Typical mitochondrial syndromes. Many patients do not show these full-blown clinical pictures and are affected by a variety of complex or partial pictures (myopathies, neuropathies, cardiomyopathies, encephalomyopathies, multisystemic diseases, and so on). The relative frequencies are indicative (specifically designed epidemiological studies are not available). mtDNA, mitochondrial DNA.

Syndrome	Relative Frequency	Typical Feature(s)	Associated Feature(s)	Inheritance	Most Frequent Genetic Findings	Treatment of Choice
Alpers syndrome	Very rare	Childhood myocerebrohepatopathy		Autosomal recessive	*POLG* mutations with secondary mtDNA depletion	Symptomatic (avoid valproate)
Autosomal dominant optic atrophy (ADOA)	Rare	Optic neuropathy (blindness)		Autosomal dominant	*OPA1* mutations	Symptomatic
Coenzyme Q10 deficiency	Very rare	Ataxia or myopathy or multi-system disease		Autosomal recessive	Various nuclear genes	Coenzyme Q10
Kearns–Sayre Syndrome (KSS)	Frequent	Ocular myopathy (ptosis, ophthalmoparesis)	Ataxia, cardiac conduction defects	Sporadic	Single large-scale deletion of mtDNA	Symptomatic
Leber hereditary optic neuropathy (LHON)	Very frequent	Optic neuropathy (blindness)		Maternal (low penetrance, higher in male smokers)	Various mtDNA mutations	Idebenone
Leigh syndrome	Frequent	Severe pediatric encephalopathy		Autosomal recessive, X-linked or maternal	Various nuclear or mtDNA mutations (e.g., m.8993T > G)	Symptomatic
Mitochondrial encephalopathy with lactic acidosis and stroke-like episodes (MELAS)	Frequent	Stroke-like episodes	Cardiac involvement, hearing loss, diabetes	Maternal	m.3243A > G	Symptomatic
Myoclonic encephalopathy with ragged-red fiber (MERRF)	Frequent	Myoclonus	Ataxia, myopathy	Maternal	m.8344A > G	Symptomatic (e.g., Levetiracetam)
Mitochondrial Neurogastrointestinal Encephalomyopathy (MNGIE)	Very rare	Gastrointestinal dysmotility	Leukodystrophy, ocular myopathy, peripheral neuropathy	Autosomal recessive	*TYMP* mutations	Liver transplantation
Neuropathy, Ataxia, Retinitis Pigmentosa (NARP)	Rare	Ataxia	Neuropathy, retinitis pigmentosa	Maternal	m.8993T > G	Symptomatic
Non syndromic hearing loss (NSHL)	Frequent	Hearing loss		Maternal	m.1555A > G	Symptomatic (avoid aminoglycosides)
Progressive external ophthalmoplegia (PEO)	Very frequent	Ocular myopathy	Myopathy	Autosomal dominant, recessive, maternal, or sporadic	Various nuclear genes with secondary mtDNA multiple deletions, various mtDNA point mutations, mtDNA single large-scale deletion	Symptomatic

**Table 3:** Revisited mitochondrial syndromes. Possible definitions for some of the most typical mitochondrial syndromes.

**Mitochondrial encephalomyopathy with lactic acidosis and stroke-like episodes (MELAS)** Patients with histological, biochemical, and/or molecular evidence of mitochondrial disease who experience stroke-like episodes. Specifically associated features (at least in MELAS due to the m.3243A>G mutation) include lactic acidosis, generalized seizures, cognitive involvement, and hearing loss.
**Myoclonic encephalomyopathy with ragged red fibers (MERRF)** A mitochondrial syndrome where myoclonus is the prominent clinical feature, and which does not meet the criteria of other well-defined mitochondrial encephalopathic syndromes, including MELAS, Leigh, and Alpers syndromes. Ataxia is a specifically associated feature, differently from epileptic seizures.
**Kearns-Sayre Syndrome (KSS) spectrum** Ophthalmoparesis and/or ptosis due to a mtDNA single large-scale deletion and at least one of the following features: Ataxia Cardiac conduction defects Cardiomyopathy Cognitive involvement Failure to thrive/short stature Hearing loss Retinopathy
**Progressive external ophthalmoplegia (PEO)** Ophthalmoparesis and/or ptosis, not fulfilling the criteria for Pearson syndrome nor “KSS spectrum” criteria or other encephalopathic syndromes. “Pure PEO”: isolated ocular myopathy. “PEO plus”: ocular myopathy with other features of neuromuscular involvement.
**Primary mitochondrial myopathies (PMM)** Genetically defined disorders leading to defects in oxidative phosphorylation affecting predominantly skeletal muscles (not fulfilling the criteria for other more complex syndromes).
